# Assessment of Primary Health Care in the Treatment of Tuberculosis in a Brazilian Locality of the International Triple Frontier

**DOI:** 10.2174/1874434601711010124

**Published:** 2017-09-22

**Authors:** Reinaldo Antonio Silva-Sobrinho, Anneliese Domingues Wysocki, Lúcia Marina Scatena, Erika Simone Galvão Pinto, Aline Ale Beraldo, Rubia Laine Paula Andrade, Adriana Zilly, Rosane Meire Munhak da Silva, Michela Prestes Gomes, Paulo César Morales Mayer, Antonio Ruffino-Netto, Tereza Cristina Scatena Villa

**Affiliations:** 1 Universidade Estadual do Oeste do Paraná, Foz do Iguaçu, Brazil; 2 Universidade Federal do Mato Grosso do Sul, Três Lagoas, Brazil; 3 Universidade Federal do Triangulo Mineiro, Uberaba, Brazil; 4 Universidade Federal do Rio Grande do Norte, Natal, Brazil; 5 Universidade de São Paulo, Ribeirão Preto, Brazil

**Keywords:** Tuberculosis, Program Evaluation and Health Services, Primary Health Care, Health on the Border, Management, Decentralization

## Abstract

**Objective:**

To evaluate the performance of Primary Health Care (PHC) in treatment of TB patients in a triple international border municipality.

**Methods:**

The present study was an evaluative survey of cross-sectional and quantitative approach conducted with 225 PHC healthcare professionals. Data was collected through a structured and validated instrument, which provided five indicators of "structure" and four indicators of "process" classified as unsatisfactory, regular or satisfactory.

**Results:**

The "structure" component was unsatisfactory for the indicator of professionals involved in TB care and training, and regular for the indicator of connection between the units and other levels of care. The "process" component was regular for the indicators of TB information, directly observed treatment and reference and counter reference on TB, and unsatisfactory for external actions on TB control.

**Conclusion:**

The "structure" and "process" components points out some weaknesses in terms of management and organization of human resources. Low frequency of training and the turnover influenced the involvement of professionals. Elements of "structure" and "process" show the need for investing in the PHC team and improving the clinical management of cases.

## 1 INTRODUCTION

Tuberculosis (TB), a “neglected disease”, is a public health problem that presented about 9 million cases in 2011, and Brazil is among the 22 countries with the highest incidence (42 cases/100,000 people). In Foz do Iguaçu, Brazil, the incidence was 47 cases/100,000 inhabitants, twice as large as the state of Paraná in 2011 [1, 2].

Variables related to the municipality's geographical features (triple international border) and population (flow and residence of foreigners, especially Paraguayan, Argentinean and Asian people) turn the control of communicable diseases into a health challenge management.

Organizing the health services (HS) is a Gordian knot for the globalization processes related to health, especially primary health care (PHC) in the borders, which offers access to residents and non-residents in domestic and foreign territory. It is therefore a complex task to establish legal instruments to support the HS exchange between countries [3].

Brazil has a universal public system with considerable financial investments aimed at improving accessibility, equity and quality of services, guided by the decentralization of technical-administrative and managerial actions [4-6].

In this scenario, one of the challenges is to geographically distribute the health care points with the resoluteness expected from HSs organized by PHC values, which reflect the degree of commitment to the goals proposed (performance) [7-10].

It is emphasized that the TB control touted by the World Health Organization is the strengthening of PHC through the improvement of health policies, financing, human resource development, equipment purchase, implementation of laboratory diagnosis and the improvement of service performance. There is also a special recommendation for the service decentralization as a strategy to deal with major treatment issues related to a service provided in a single health unit. Among them: patients access to diagnosis and treatment; daily following of the drug administration for six months; active search of the patient’s relatives; periodical clinical exams; and clinical management of the intercurrences [11].

The decentralization of the TB control actions have been encouraged, in Brazil, by the National Program of TB Control since it broadens the access to health services, promotes the patients adhesion to the treatment and strengthens the bond between the patient and the health service. Through the decentralization, health professionals are expected to have better conditions of developing many actions such as: active search of sick people with respiratory symptoms, requests of diagnostic exams, information recording, assisted treatment, patients follow-up and contact exams [12].

However, there are obstacles related to PHC regarding access to diagnosis, exams, delay in starting the treatment, Directly Observed Treatment (DOT) and the management of multidrug-resistant cases, caused by incomplete decentralization and limited political commitment, disability integrated approach and lack of permanent actions [7, 13].

Brazilian studies on TB control in PHC show mixed results as an increase in the number of respiratory symptoms, examined contacts and smears. Paradoxically, there is underperforming access to diagnosis, lack of involvement and coordination between managers, vertical management and high turnover of professionals – what contributes to poor performance of TB control in the PHC [4, 14-17]. Although people suffering from TB usually first resort to the PHS to treat their condition, these services have not been effective, considering the delay in the diagnostic and consequently in the treatment as well, demonstrating the lack of integration between the health services [18].

The organization of health care systems, the integration and the decentralization policies occur based on the economic and social dynamics of each country [6]. Considering that: a) the TB incidence in Foz do Iguaçu is higher than the already alarming national average; b) the local health assistance network deals with an itinerant and multicultural public; c) the decentralization of health services for TB patients has taken place in the city only one year ago; and d) that many difficulties in the decentralization process of TB treatment were detected in another Brazilian cities, the present study was designed to evaluate the performance of PHC in TB control in a triple international border municipality in its first year following the decentralization of the disease control.

## 2 METHODS

### 2.1 Design and Scenario of the Study

This is a survey type and cross-sectional study carried out in Foz do Iguaçu, a Brazilian city located in the triple border Brazil, Paraguay and Argentina, with a population of 256,088 in 2011 [19].

Their health care is provided by 28 Units of Primary Health Care (UPHC) with 34 Family Health Teams (FHT), six Emergency Services, Municipal and Private Hospitals and medical specialties reference center.

TB control actions are carried out inside the UPHCs, with the help of Matrix Teams (MT) consisting of three nurses, a nursing technician and a physician, that give support in assistance and pedagogical issues in TB. DOT is done by a UPHC professional that can be a Community Health Agent (CHA), a nurse or a nursing assistant/technician, and for the most serious cases and/or comorbidities, the MT.

The FHTs work six hours daily from Monday through Saturday, so there is always at least one team in the unit during the day. The UPHCs have hardware, internet and software for operation of the electronic medical record.

UPHC health care professionals are responsible for longitudinal actions in time. A physician makes the diagnosis and indicates clinical treatment, conducts monthly/intercurrence consultations and determines the discharge by cure. The nurse is the managerial organizer who assists the treatments, registers epidemiological and management data with the support of nursing auxiliary/technicians and CHA.

In the units where there are no doctors/nurses or where they have not adhered to TB treatment, the MT performs the service with the UPHC teams in an attempt to train/raise awareness among them.

### 2.2 Sample Calculation and Study Subjects

The population of the study was comprised of healthcare professionals working in UPHCs. The number of professionals working in UPHCs registered in the National Register of Health Facilities was obtained and the duplicate records were deleted.

For the calculation of the sample size, the total population of professionals (518) who worked in UPHC served as reference also with these parameters: sampling error of 0.05; confidence interval of 95% and P (population percentage) of 50%. The equation $n_{0}=\frac{p.\left(1-p\right).Z\alpha^{2}}{e^{2}}$was used to obtain the minimum sample size, which was corrected (since it is a finite population) to the total population of professionals using the equation, $n=\frac{n_{0}}{1+\left(n_{0}-1/N\right)}$ resulting in a number of 225 professionals to be interviewed. The amount of doctors, nurses, nursing assistants/technicians and CHA to be interviewed was defined by proportional sharing. The health units were selected through simple random sorting according to the sanitary health district (Eastern, Western, Northern, Southern, Northeastern) to assure territorial representativeness.

The order of the interviews was defined by sortition (simple random sampling) of the UPHC and all the professionals identified in the unit were interviewed. Sortitions were conducted until the required number of individuals in the sample size calculation was reached.

The 225 health care professionals interviewed in the 23 UPHC in 2011 met the inclusion criteria of having followed a TB case in the past six months.

### 2.3 Instrument and Data Collection

It was used a structured questionnaire, which was elaborated and validated by the Group of Epidemiological-Operational Studies on Tuberculosis (GEOTB) of the Brazilian Tuberculosis Research Network (REDE-TB), which contains performance evaluation variables of an PHC in monitoring TB cases [16].

The instrument comprehended 78 questions. Variables were divided into four blocks: 1) health unit identification; 2) applicant identification; 3) evaluation of the *structure* components (human resources, physical resources, and service organization); and 4) evaluation of the *process* components. The *structure* component represents the available resources for the assistance occurrence and the *process* component consists of a group of actions performed by and between the professionals and the clients including the attributes of continuity, coordination, way of assisting the patient, and teamwork, conforming the attention offered with the attention actually received [20, 21].

Responses occurred according to a dichotomic scale or multiple choice of single option selection. The evaluations were categorized into unsatisfactory, regular, or satisfactory according to the criteria presented further.

Visits to the health units were scheduled by phone. Interviews were conducted by four undergraduate students from the nursing course, which received a systematic training. Subjects were interviewed individually in the health units they worked, in a separate room, to ensure privacy. Each interview lasted in average 20 minutes.

The study was approved by the Ethics Committee of the State University of Western Paraná, Protocol No. 166/2011.

### 2.4 Data Analysis

Descriptive techniques were used in the construction of HS performance assessment indicators, as follows: 5 related to *structure* (professionals involved in TB care, training, access to recording instruments, connection of the HS with other levels of care and availability of inputs); and 4 related to *process* (information about TB, DOT, external action to control TB and reference and counter reference for other HS).

To build the indicators, the results of the multicenter project in which this study is integrated were used as a reference [16]. A reference indicator accounted for μ=P average (P equals the proportion of the studied characteristic) and standard deviation $\left(\sigma_{p}=\sqrt{\frac{P\left(1-P\right)}{n}}\right)$ of the questionnaire items that made up each indicator.

Based on the reference indicators, an indicator was calculated for each HS and for the city by using the standardized value $z=\frac{P_{i}-\mu }{\sigma_{p}}$ (P_i_, proportion of each HS with the characteristics studied and/or the proportion of the city with the characteristics studied; μ, reference indicator and σ_p_, standard deviation) considering the approximation to the binomial distribution to normal distribution. The Z=1 value was taken into account, that is, a standard deviation for the differences observed between P_i_ and μ. Thus, the UPHCs evaluated with indicators bellow -1 (z= <-1) did not reach the average value and were found to be unsatisfactory, those values between -1 <z <1 reached average value and were considered regular, and those above 1 (z> 1) were considered above the medium value, thus satisfactory.

## 3 RESULTS

From the 28 PHS units of the city 23 participated in the study, a total of 225 health professionals were interviewed.

Results from the human resources item, in the *structure* indicator, showed low involvement of the team members without academic degree in patient assistance, due to low investment in TB capacitation for this group of professionals. Conversely, the items “*physical resource”*’ and “*service organization*”, both presented elevated rate of affirmative responses (Table **1**).

Regarding the *process* indicator, the low levels reported in the evaluation were mainly influenced by deficiencies related to: DOT offering at work; TB educational actions aimed at the community; monthly requests of smear control (bacilloscopy); and finally to the counter reference of the information relating to medical appointments outside of that health service (Table **1**).

The UPHC *structure* component showed unsatisfactory value for professional indicators involved in TB care and training Fig. (**1**), regular value for the articulation indicators of the UPHC with other levels of care, and satisfactory value for indicators of access to registration instruments and availability of inputs (Fig. **2**).

**Fig. (1):**
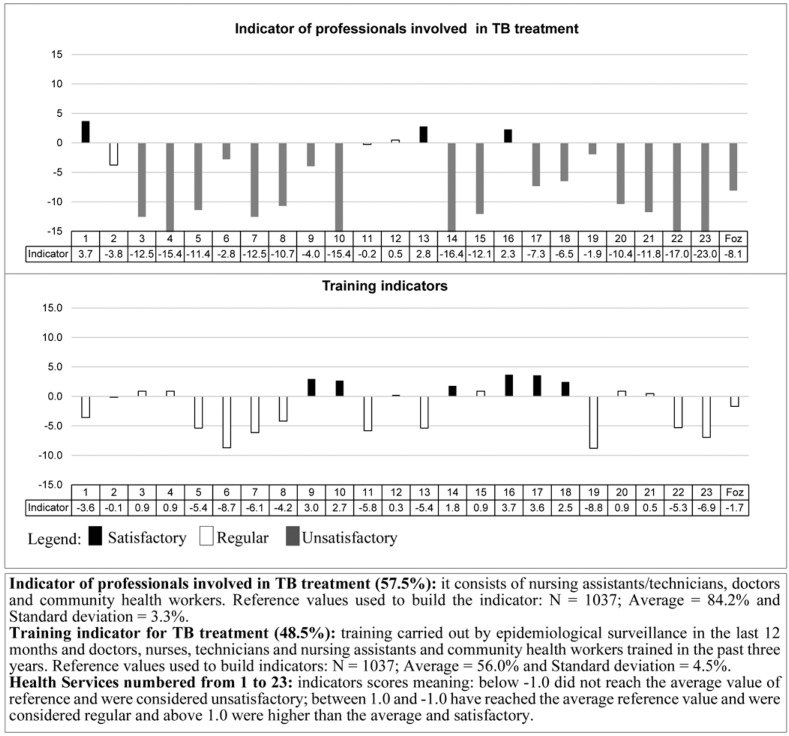
Structure indicators of the Primary Health Care Units in terms of human resources - Foz do Iguaçu-PR 2011.

**Fig. (2):**
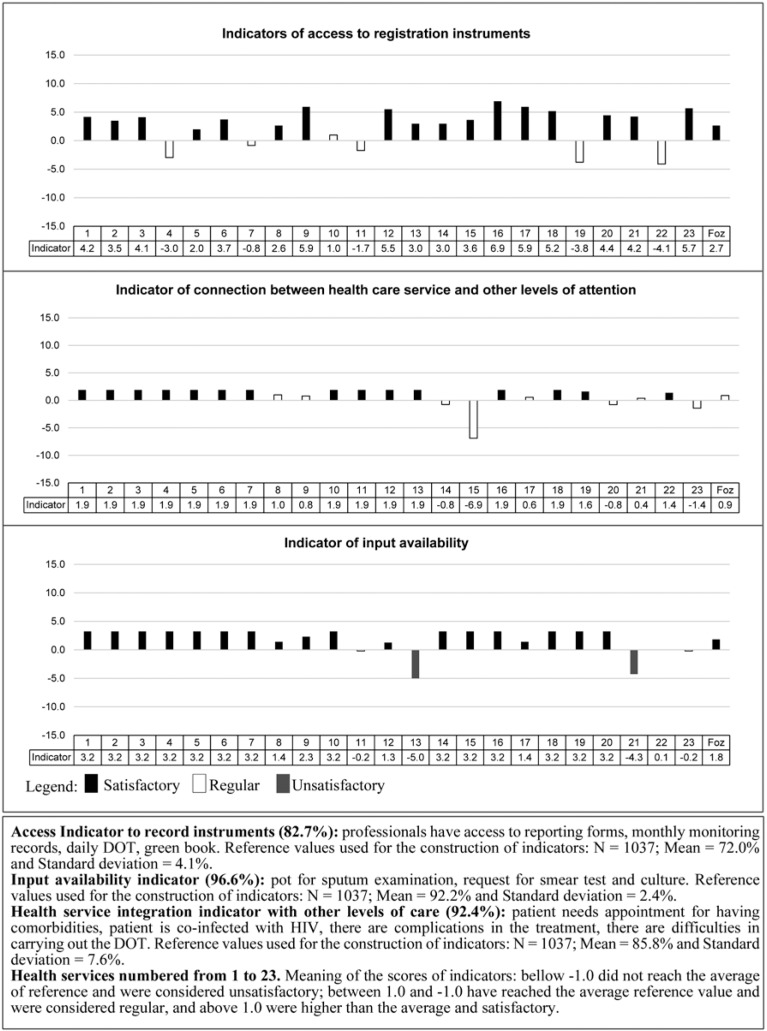
Structure indicators of the Primary Health Care Units in terms of physical resources, Foz do Iguaçu-PR 2011.

Regarding the UPHCs, 18 (51.4%) did not reach satisfactory values ​​for professional indicators involved in TB care, 10 (43.5%) for the training indicator, 4 (17.4%) for the indicator of access to registration instruments and 2 (8.7%) for the indicator of availability of inputs and coordination with other levels of care (Figs. **1** and **2**).

For the *process* component Figs. (**3** and **4**), the city had regular value for the indicators of information about TB, DOT and reference/counter reference, and insufficient value for the indicator of external actions to control TB. The UPHCs that had unsatisfactory values ​​for *process* indicators were: 4 (17.4%) for information on TB, 7 (30.4%) for DOT, 22 (95.7%) for external actions and 5 (21.7%) for reference/counter reference.

## 4 DISCUSSION

In assessing the UPHCs on the *structure* component (human resources), there was lack of integration and interaction between professionals, as not all of them took part in the TB assistance work, mainly nursing assistants/technicians. Therefore, the treatment occurred without a care plan involving all the staff or clients. However, one must consider the challenge to organize the work *process*, considering the PHC demands in terms of services offered and the complexity of treatment and care provided [9].

To control TB in the PHC, the maintenance of complete teams and the involvement of professionals in the healthcare plan is essential because care must be developed by multi-professional teams [22] given the positive connection between high treatment adherence and UPHC with complete teams (nurse, physician, nursing auxiliary/technicians and CHA) especially when the nursing assistant is involved [23].

The *training indicator* showed that in the last three years there has been no theoretical and practical training on TB for the teams. The ones favored by the managers were: nurses (74.7%) and physicians (59.1%), which reveals some emphasis on the training of professionals with higher education [24]. This training was timely, stimulated by the State Coordination of TB, not a preparation strategy before a decentralization of assistance for the PHC. However, as a component of the decentralization plan, an MT was stablished for shared organization/coordination of the working *process* for clinical support, supervision and training of the teams [25].

However, during MT visits, UPHC professionals (except nurses) did not devote time to discuss about TB assistance. These difficulties may be related to the workload and disability in the preparation of an MT for “systematic exchange of knowledge among the various specialties and professions”, *i.e.* it was not prepared to “identify epistemological obstacles and the *structure* of services”, which are requisites to an MT action [26].

Thus, a limited integration between UPHC and MT was identified so that there was no satisfactory incorporation of the disease control recommended for PHC, except the partial completion of the DOT. It is highlighted the need to incorporate systematic/permanent educational processes, as in transboundary areas, ethnic and cultural diversity makes TB assistance more challenging.

Indicators of *access to recording instruments and availability of inputs* showed that there has been preparation of the physical features of PHC, so they were accessible in the UPHC. The variables that made up these two indicators have been well evaluated, with percentages above 83% (except access to the TB Case Registration and Monitoring Book, 57.8%).

It happens that the managers designate a nurse as the responsible for the registration of data [27], so the other members of the team are not encouraged to register their actions in the TB Case Registration and Monitoring Book and are even unaware of it. The PHC Brazilian Policy says that this action means team liability [28]. However, it was found the existence of a division of tasks concerning the records; doctors wrote down the clinical data from medical records and nurses generated epidemiological data (TB Case Registration and Monitoring Book and daily control of the DOT) and management (data consolidation reports).

A nurse develops assistance connected to programmatic policies in the PHC, which in most cases are administrative responsibilities related to the organization of the work *process*, infrastructure and planning [29]. Due to these features, he/she is recognized as an organizer and executor of management and collective actions, however they can lead them to a centralized approach, thus discouraging the acquisition of autonomy and responsibility of other members of the team [30].

The indicator of *connection of the health service with other care points* was regular, a challenging situation to PHC because of the lack of places and the delay in accessing specialists. In this case, the assessment was possibly less negative because of the presence of the MT, which is a reference to speed the access to diagnostic tests and solve clinical and operational complications.

The public health policies present the PHC strategy as a reorganization of the health care model characterized by the provision of comprehensive health actions, by defining responsibilities of the HSs with agreements to ensure complementarity and interdependence [31]. In view of this, the connection within the UPHC or other care points is in a technology that enhances the outcomes of TB care.

Considering the territorial features, it becomes urgent to connect with the local agenda of the health systems from Paraguay and Argentina (objective laid down in the Mercosur) [3]. People of these countries seek diagnosis and medication for TB in Brazilian UPHCs and return to their homes, lacking monthly clinical monitoring while our PHC lacks information about the progress of their treatment until some spontaneous return. It should be noted here, the essence of public policies to address the border areas that transcend national boundaries, the regional integration projects and the municipal programs that consider the vulnerabilities and peculiarities of these regions.

Regarding the attention provided (*process*) during their treatment, the *TB information* indicator had regular evaluation. It is believed that the visit of a MT in the UPHC when there may be new cases might have influenced this indicator, as the amount of information recommended by the Ministry of Health [32] was informed to the patient by the MT with the PHC professionals.

The lack of information is associated with poor adherence to treatment and care failure. On the other hand, when professionals give clear information, it is expressed their attention to clinical protocols and therapeutic guidelines endorsed for the management of TB, thus indicating organic quality in the work *process* [4, 33].

About the DOT indicator, field observation does not corroborate the findings, because invariably, the drug is dispensed for self-administration for periods of 10 days. In Brazil, the DOT is supposed to be held from Monday through Friday in the attack phase and at least three times a week in the maintenance phase, supervised by a health professional at home, at the HS or at the patient's workplace [34].

A study conducted in Brazil showed a variation in the DOT where municipalities of greater FHS coverage showed a worse performance. This study highlights the difficulties that UPHCs have to perform actions focused on family and community, and the appointments are scheduled to occur within the UPHC, resuming the individual and biomedical model [4].

The indicator of *external actions* showed that extramural activities related to TB are almost nonexistent and possibly because of the care model still in force, the individual is oriented to acute situations [9]. Moreover, issues related to the PHC organization such as workload reduction to six hours daily, presence of incomplete teams, medical and/or nursing absence/turnover due to temporary contracts and the presence of physicians serving more than one UPHC are aspects that interfered in the city health care activity program, thus impairing the control of health priorities such as TB and weakening the reorganization of PHC, plastering the current care model.

This scenario makes the TB work *process* incipient and poorly expressive, since the difficulties to develop health education and home visits are linked to lack of teamwork and continuous professional training, insufficient human resources, and weak coordination between community and professional and public management of the health system [34, 35].

Given the mobility of people and families due to their commercial links, social rights and health, seeking better living conditions in the tri-border region, the provision of educational activities can be a powerful health surveillance tool, and when it comes to an infectious disease such as TB, raising awareness about it is fundamental to control it [36].

Considering the existence of electronic medical records in public service network, which allows for appointments and tests, the indicator of *reference and counter reference* should have a better performance. In UPHC, examinations/appointments are scheduled online and additional written information is provided for delivery in service referred. Municipal services for medical, laboratory and radiological specialty can register/attach the results in the electronic medical record, but there has not been significant adhesion to this technological tool to contribute to the exchange of information between professionals and the HS, being the variable that had the worst performance in this indicator.

It is important to organize the work *process*, in order to articulate the PHC and its diagnostic support services. The reference/counter reference system is strategic for the development of a network that ensures complementarity and interdependence between different services [9].

The HS reorganization that determines TB control exclusively at the UPHC, has altered the dynamics of work in a system that was not prepared, given the presence of incomplete PHC teams (insufficient number of professionals), high staff turnover due to their way of hiring (outsourcing). Thus, it seems that the teams did not understand the dynamics of decentralization, failing to use the management tools, such as the computerized system.

The observed asymmetries between the indicators of *structure* and of *process,* seem to be a reflex of fragmented health systems channeled to the care of acute conditions and to the acutization of chronic conditions, even though they are under the guidelines of APS. One of the tenets of the family and community centered care is the knowledge/articulation/commitment of the health professionals themselves and with the community. In support of this rationale the World Health Organization highlights the emphasis on “interprofessional education and collaborative practice” to its member nations once the evidences indicate these technologies strengthen APS-oriented health systems, being decisive in dealing with global challenges such as HIV/Aids and tuberculosis epidemics [37].

The present evidences indicated that organizational management and effective planning are proportionally relevant dimensions of adequate health service delivery. Specifically, for the APS in the location researched the results point to the need of improvement in permanent education and in the articulation with other health attention services through management contracts as suggested alternatives to deal with the fragility of the clinical handling of individual, family and community centered multiprofessional care.

In what concerns to research issues, the greatest contribution of the present study is the viability of the evaluative method implemented. The research instrument was built up according to international guidelines and aims for the tuberculosis care, to scientific evidences, and to APS values [16], presenting itself as a relevant tool to assess the performance of the APS services in the control of TB and stressing the importance of the operational research as a support for the health care management.

The possibility of an information bias is pointed out as a limitation, given the negative aspects that might have been omitted. It should be noted that the information reached reflects the perspective of professionals, and it is recommended for future research to include other actors involved in TB care system. The results need to be considered with discretion when generalized to other municipalities.

## CONCLUSION

The evaluation of the TB control pointed partial strongholds in relation to the *structure* of the UPHCs. Professionals had access to recording instruments and supplies. Items linked to human resources showed the worst performance. The greatest weaknesses were manifested by the *process* component, thus suggesting deficiency in clinical management of home visiting and educational activities aimed at the community. The PHC teams need to improve the organization of care, by optimizing the existing *structure*, in order to continue the treatment to promote longitudinal care. They need to understand it as an innovation and restructure practice to rescue the expanded concept of health and disease that encompasses both family and community through the involvement of the entire multidisciplinary team.

The assessment of the tuberculosis control program according to different sets of indicators allowed punctual critics of the specific actions that did not work properly. Although the aims and the material resources were coherent, permanent interprofessional education based on the APS principles is still needed. Concomitantly, it is fundamental to monitor the outcomes/impact of the tuberculosis care through evaluative operational researches contemplating the perspectives of the healthcare professionals, managers and users.

## Figures and Tables

**Fig. (3):**
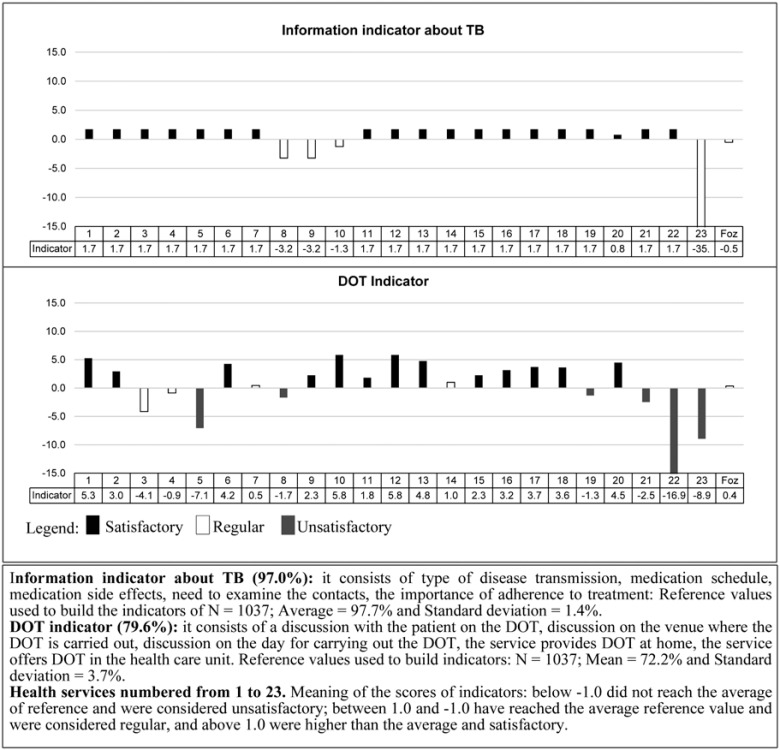
Process Indicators of Primary Health Units in terms of information about TB and directly observed treatment (DOT), Foz do Iguaçu-PR 2011.

**Fig. (4):**
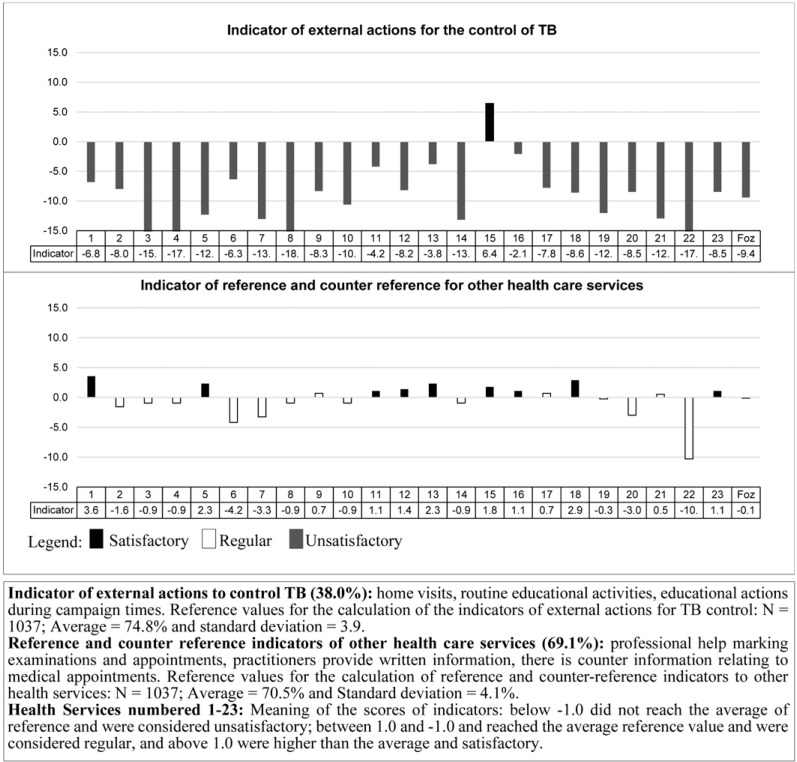
Process indicators of Primary Health Units in terms of external actions for the control TB and reference and counter, Foz do Iguaçu-PR 2011.

**Table 1:** Proportion of the questionnaire items of the structure and process components.

	*Items of the structure* *component* *questionnaire* *-* *human resources*	%
7.2	Does the HS have a nurse assistant/technician involved with the care of TB patients in this service?	35.6
7.3	Does the HS have a doctor involved in the care of TB patients in this service?	76.4
7.4	Does the HS have a community health worker involved with the care of TB patients in this service?	66.7
9.1	Over the past three years, have nurses been trained in TB health care? Does the HS have a community health worker involved with the care of TB patients in this service?	74.7
9.2	Over the past three years, were the nursing assistants/technicians trained in TB health care?	11.6
9.3	In the last three years, have doctors been trained in TB health care?	59.1
9.4	Over the past three years, have the CHAs been trained in TB health care?	26.2
10	In the last 12 months was there any training in TB held by the Health Epidemiological Surveillance for this service?	72.9
	*Items of the structure* *component* *questionnaire* *-* *physical resources*	
16.1	Do the professionals have access to report forms?	92.0
16.2	Professionals have access to medical records.	96.4
16.3	Professionals have access to monthly follow-up treatment files.	90.7
16.4	Professionals have access to DOT daily file.	83.6
16.5	Professionals have access to the Green Book.	57.8
17.1	The HS has pot for sputum examination.	96.0
17.2	The HS has smear application.	97.3
17.3	The HS has culture application.	97.8
	*Items of the structure component questionnaire - service organization*	
23.1	There is a connection between this service and other levels of care when the patient needs appointments for having other comorbidities (diabetes, hypertension, psychiatric disorder)	94.7
23.2	There is a connection between this service and other levels of care when the patient is co-infected with HIV.	95.1
23.3	There is a connection between this service and other levels of care when there are complications in the treatment.	93.3
23.4	There is a connection between this service and other levels of care when there are difficulties in carrying out the DOT.	92.0
	*Items of the process component questionnaire*	
29.1	Information is passed about TB transmission.	98.2
29.2	Information is passed about TB medication schedule.	98.2
29.3	Information is passed about TB side effects.	98.2
29.4	Information is passed about TB and the need to examine contacts.	97.3
29.5	Information is passed about the importance of adherence to TB treatment.	98.2
30.1	There is discussion with the TB patient on the implementation of Directly Observed Treatment (DOT).	89.8
30.2	There is discussion with the TB patient on the location of the DOT.	89.3
30.3	There is discussion with the TB patient on the day the DOT should be carried out.	88.9
32.1	This health service provides DOT at home.	72.4
32.2	This health care facility offers DOT at the SUS facilities.	63.1
32.3*	This health care facility offers DOT at work.	2.2
33.	Does this health care facility offer Home Visitation (HV) for priority cases of TB (patients with smear +, HIV positive, alcoholics, drug addicts)?	69.3
34.1	Are the TB educational actions aimed at the community carried out routinely?	10.2
34.2	Are the TB educational actions aimed at the community held during campaigns seasons?	28.4
36.*	Does this health care service request smear control monthly?	27.6
40.	When the patient is referred to other health services, do professionals help scheduling the examinations and appointments?	93.8
41.	When the patient is referred to other health services, do professionals provide written information (reference form, examination results, letters, etc.) to be handed in to that service?	85.8
42.	Is there any counter reference of the information relating to medical appointments outside of this health service?	21.8

*Items 'this health care unit offers DOT at work' and 'this health service requests smear monthly control' have not been validated. However, they were selected for presentation.
